# Preparing for Ebola: Strengthening Nursing Readiness in the United States

**DOI:** 10.1002/nop2.70797

**Published:** 2026-08-30

**Authors:** Benjamin Lindsay

**Affiliations:** ^1^ Senior Nursing Professional Development Specialist in Adult Critical Care Rochester New York USA

Emerging infectious diseases remain an ongoing global public health challenge, requiring healthcare systems and nurses to continually adapt their preparedness and response efforts. This editorial focuses on the United States' health preparedness. Healthcare professionals encounter infectious diseases transmitted through contact, droplet, and airborne routes, underscoring the importance of maintaining preparedness for a range of infectious threats. Ensuring readiness for even the most contagious and deadly infectious diseases requires coordination among public health organizations and healthcare systems nationwide. The National Emerging Special Pathogens Training and Education Center (NETEC) is one organization that supports emergency preparedness for High‐Consequence Infectious Diseases (HCID), such as Ebola (National Emerging Special Pathogens Training and Education Center 2025). Although rare in the United States, recent Ebola activity highlights the importance of maintaining healthcare readiness.

Nurses should understand HCIDs and the principles of preparedness to support safe and effective responses. HCIDs are characterized by factors including severity, transmissibility, incubation period, environmental stability, and the availability of effective treatments or preventive measures (National Emerging Special Pathogens Training and Education Center 2025). Understanding these characteristics helps healthcare systems and public health agencies develop appropriate responses and enables nurses to determine appropriate isolation precautions, PPE selection, and escalation procedures.

Ebola virus disease (EVD) is a severe and potentially fatal viral disease. In May 2026, the Democratic Republic of the Congo (DRC) declared an outbreak of Bundibugyo virus disease, with subsequent cross‐border transmission to Uganda (World Health Organization 2026c). According to the World Health Organization (WHO), three viruses are known to cause large Ebola disease outbreaks: the Ebola virus, the Sudan virus, and the Bundibugyo virus (World Health Organization 2025). The WHO declared the outbreak a Public Health Emergency of International Concern (PHEIC) due to its severity and potential for international spread (World Health Organization 2026c). Case fatality rates in past Ebola outbreaks have ranged from 25% to 90%, with an average case fatality rate of approximately 50% (World Health Organization 2025). Although outcomes vary depending on the virus species, access to healthcare, and the timeliness of treatment, early recognition and supportive care significantly improve survival (World Health Organization 2025; Centers for Disease Control and Prevention 2026).

Ebola virus disease, first recognized in 1976, is thought to have been introduced into human populations through zoonotic transmission. Subsequently, it spreads from person to person through direct contact with the blood or other bodily fluids of infected individuals or with contaminated materials (World Health Organization 2025). After an incubation period of 2 to 21 days, with an average of 8 to 10 days, initial symptoms often appear suddenly (World Health Organization 2025). These include fever, intense weakness, muscle pain, headache, and a sore throat (World Health Organization 2025). As the disease progresses, patients may experience vomiting, diarrhoea, a rash, and impaired kidney and liver function. In severe cases, both internal and external bleeding can occur.

Although treatment options differ by Ebola virus species, advances in clinical management have improved outcomes. Two monoclonal antibody therapies are approved in the United States for Ebola disease caused by Orthoebolavirus zairense, and the Ervebo vaccine is licensed for the prevention of disease caused by this virus. These countermeasures are not currently licensed for Bundibugyo virus disease (Centers for Disease Control and Prevention 2026; World Health Organization 2025, 2026a). For all forms of Ebola disease, early recognition and optimized supportive care, including fluid and electrolyte management, hemodynamic support, oxygenation, nutritional support, and treatment of complications and secondary infections, remain essential components of clinical management (Centers for Disease Control and Prevention 2026; World Health Organization 2025).

When Ebola is suspected based on signs and symptoms or known exposure, the individual should be isolated (Centers for Disease Control and Prevention 2026). Known exposure, also known as Patients Under Investigation, refers to contact with an individual with a confirmed or suspected infection without appropriate protective equipment. Immediately engage infection prevention, organizational leadership, and appropriate public health authorities in accordance with organizational protocols. Follow current CDC and organizational guidance for PPE selection, donning, and doffing, with trained personnel providing oversight when available (Centers for Disease Control and Prevention 2026). Minimize the number of healthcare workers entering the patient's care environment and use dedicated equipment when appropriate. Ebola should not be assumed to be the diagnosis; evaluate for other causes of compatible illness while Ebola testing and public health assessment proceed.

As we reflect on past infectious disease outbreaks locally and globally, from norovirus to HIV, nurses have consistently provided care despite uncertainty, stigma, and evolving scientific knowledge. During the HIV epidemic of the 1980s, nurses cared for patients amid fear, stigma, uncertainty, and limited understanding of the disease. More recently, the COVID‐19 pandemic affected healthcare systems and communities worldwide. Throughout these public health crises, nurses have remained committed to providing safe, compassionate, and ethical care while balancing their responsibility to protect both their patients and themselves. In times of crisis, healthcare professionals are often portrayed as heroic because of the inherent risks of their work, even though risk is always part of their roles. Nursing practice is grounded in professional ethical standards that emphasize compassionate care, respect for human dignity, patient advocacy, confidentiality, and the promotion of health and well‐being (American Nurses Association 2025).

The COVID‐19 pandemic highlighted the consequences of supply chain shortages, PPE conservation efforts, and rapidly changing guidance, contributing to fatigue and burnout among healthcare workers (Billings et al. 2021). Preparedness should therefore address not only physical safety but also psychological health, including access to clear communication, leadership support, mental health resources, and mechanisms for reporting concerns without fear of retaliation. When preparedness gaps are identified, nurses can advocate through established organizational channels by reporting supply shortages, participating in preparedness committees and drills, identifying gaps in policies or competencies, and engaging leaders in mitigation planning. Protecting healthcare workers is an essential component of patient safety and organizational preparedness.

The 2014–2016 West Africa Ebola epidemic demonstrated the significant occupational risks faced by healthcare workers caring for patients with Ebola (Raven et al. 2018). The 2026 Bundibugyo virus disease outbreak in the DRC further demonstrates that these risks remain relevant. As of July 1, 2026, the WHO reported 102 confirmed cases among health and care workers in the DRC, including 25 deaths (World Health Organization 2026b). These findings reinforce the importance of infection prevention and control, appropriate PPE, training, and organizational preparedness.

Emergency and highly infectious disease preparedness is a shared responsibility of healthcare organizations, nurses, and the communities they serve. Preparedness is not the responsibility of individual nurses alone; it requires sustained investment and support from institutions, governments, and public health agencies (Raven et al. 2018). Organizations including the WHO, the U.S. Department of Health and Human Services, the Centers for Disease Control and Prevention (CDC), and NETEC provide guidance, education, and support for special pathogen preparedness. Healthcare systems should use these resources to identify gaps in infrastructure, policies, training, and workforce readiness.

Healthcare organizations should strive to have an established infrastructure, policies and procedures, and a culture of readiness to manage suspected or confirmed special pathogen incidents across their healthcare systems. Infrastructure may include, but is not limited to, a laboratory equipped to test special pathogens to ensure early diagnosis, an air filtration system, a secure biocontainment unit, PPE supplies, a robust training process, interprofessional collaboration, ongoing competency validation, simulation, and regular clinical practice drills.

Healthcare systems that invest in preparedness, clear communication, and support for the frontline workforce are better positioned to respond effectively to high‐consequence infectious disease events. Emergency preparedness and readiness are not individual sacrifices; they are a shared ethical responsibility to use current knowledge to inform best practices for frontline staff who support patients, as well as nurses working across healthcare settings and within the broader community. Healthcare organizations have a responsibility to support the health and well‐being of the communities they serve, particularly during public health emergencies. When organizations demonstrate this support through policies, equipment, and spaces, it reassures nurses that they are protected and valued, fostering a sense of safety and confidence in their work.

Ebola preparedness reflects a broader commitment to patients, communities, and public health. Sustained investment in preparedness, education, infrastructure, and workforce support is essential to ensuring that nurses remain equipped to respond safely and effectively to future high‐consequence infectious disease events. Readiness is not a reflection of fear or heroism; it is a reflection of professional commitment to our patients, our colleagues, our communities, and the future of nursing.

## Author Contributions

**Benjamin Lindsay:** conceptualization, resources, writing – review and editing.

## Funding

The author has nothing to report.

## Conflicts of Interest

The author declares no conflicts of interest.

## Data Availability

Data sharing not applicable to this article as no datasets were generated or analysed during the current study.
